# Independent association of weight-adjusted waist index with asthma in U.S. adolescents: Mediating roles of eosinophil percentage, total cholesterol, and HDL cholesterol

**DOI:** 10.1371/journal.pone.0328796

**Published:** 2025-07-31

**Authors:** Lei He, Yingxia Deng, Xiaohua Liu, Yao Wang, Jing Tang

**Affiliations:** Department of Pediatrics, Shuangliu Maternal and Child Health Hospital, Chengdu, China; Yonsei University Medical Center: Yonsei University Health System, KOREA, REPUBLIC OF

## Abstract

**Background:**

Obesity is a risk factor for asthma in adolescents. “Although body mass index (BMI) is the most widely used metric for assessing obesity, it has several limitations. The weight-adjusted waist index (WWI) is a novel central obesity indicator and accurately reflects body composition. We aimed to explore the association of WWI with asthma in adolescents using NHANES 1999–2020 data.

**Methods:**

WWI was calculated based on waist circumference (cm) divided by the square root of weight (kg). Current asthma status in adolescents was determined based on participants’ self-reports. Multivariate logistic regression analysis, restricted cubic spline (RCS) analysis, mediation analysis, and stratified analysis were used to comprehensively explore this association.

**Results:**

A total of 15,796 adolescents were included. In the fully adjusted model, WWI was positively associated with current asthma in adolescents (odds ratio 1.252, 95% confidence interval 1.125–1.392, p = 0.0001). Participants in the highest WWI quartile (Q4) showed a 54.5% higher prevalence of asthma compared to Q1 (p for trend = 0.0007).. RCS modeling indicated that the association was linear. Mediation analyses indicated that blood eosinophil percentage (EOS%), total cholesterol (TC), and high-density lipoprotein cholesterol (HDL-C) partially mediated this association by 9.89% (p < 0.0001), 7.47% (p = 0.048), and 8.24% (p = 0.044), respectively. This association was independent of BMI, and BMI also did not significantly interact with this association (p for interaction = 0.682).

**Conclusions:**

WWI was linearly and positively associated with the prevalence of current asthma among U.S. adolescents, independently of BMI. EOS%, TC, and HDL-C partially mediated this association.

## 1. Introduction

Asthma is a heterogeneous disease characterized by chronic airway inflammation and airway hyperresponsiveness, with clinical manifestations of recurrent episodes of wheezing, coughing, shortness of breath, and chest tightness with reversible expiratory airflow limitation [1]. Asthma is one of the most common chronic noncommunicable diseases and continues to pose a significant global health burden, particularly among childhood and adolescence [2,3]. The global prevalence of current asthma symptoms in this age group is approximately 10%, and it continues to pose a significant health and economic burden in countries like the United States [4–6]. Uncontrolled asthma significantly reduces adolescents’ quality of life, resulting in limited daily activities, sleep disturbances, academic absenteeism, and psychological distress [7–10]. Over- and under-diagnosis of asthma in adolescents remains a significant challenge [11]. Early identification and intervention in asthma occurrence through the recognition of modifiable risk factors and the implementation of personalized prevention strategies are important.

Obesity has been identified as a significant risk factor for asthma, and accumulating evidence suggests that relationship between the two may represent a distinct phenotype of asthma [12]. While many studies suggest that obesity increases the risk and severity of asthma in adolescents, the evidence is not entirely consistent. Some observational studies have found no significant link between obesity defined by body mass index (BMI) and asthma outcomes in adolescents [13–16]. These inconsistencies may stem from the inherent limitations of BMI, which, despite being a common metric, cannot distinguish between fat and muscle mass or characterize body fat distribution [17]. Consequently, indicators that better reflect central obesity and body composition may more accurately capture the inflammatory responses and metabolic dysregulation relevant to asthma [18,19].

To address the limitations of BMI, the weight-adjusted waist index (WWI) was proposed as a novel anthropometric indicator of central obesity [20]. The WWI standardized waist circumference (WC) by weight, not only highlighting the predominance of central obesity, but also weakening the association with BMI [21]. Importantly, recent evidence demonstrates that WWI is a strong predictor of body composition, correlating positively with fat mass and negatively with muscle mass [22–24]. Cross-sectional studies in adults have already linked higher WWI to an increased prevalence of asthma, a later age of asthma onset, and higher mortality rates in individuals with asthma, highlighting its potential clinical utility [25,26]. In addition, there was a significant positive correlation between asthma duration and WWI [27].

Despite these promising findings in adults, it remains unknown whether WWI is independently associated with asthma prevalence among adolescents. Addressing these knowledge gaps could help reveal whether WWI is a modifiable risk factor for asthma in adolescents and serve as a potential target for intervention. Therefore, this study utilized data from the National Health and Nutrition Examination Survey (NHANES) to investigate the association between WWI and asthma prevalence in U.S. adolescents. We aimed to determine if this relationship is independent of BMI and to explore the potential mediating roles of inflammatory and metabolic markers.

## 2. Methods

### 2.1. Data collection process

We extracted data from NHANES, a nationally representative cross-sectional survey conducted in the United States between 1999 and 2020. Adolescents aged 12–19 years were included. Participants were eligible if they had complete information on asthma status, anthropometric measures required to calculate WWI, and relevant covariates including socioeconomic status, dietary intake, prescription medication use, and household smoking exposure. Individuals were excluded if any of these key variables were missing.

### 2.2. Study design and population

NHANES is an ongoing national cross-sectional survey led by the National Center for Health Statistics (NCHS) of the U.S. Centers for Disease Control and Prevention (CDC) to assess the health and nutritional status of community-dwelling populations. Since 1999, NHANES has employed a continuous survey format, with a separate cycle every two years, to survey a nationally representative sample of approximately 5,000 participants to collect data from interviews, physical examinations, and laboratory tests. NHANES utilized a stratified multistage probability sampling strategy to ensure that the results reflected the true proportion of the national population. All survey protocols were approved by the NCHS Ethics Review Board, and all participants signed written informed consent prior to the survey. The process strictly adhered to the ethical standards of the Declaration of Helsinki and subsequent revisions for human research.

### 2.3. Assessment of WWI

WWI was calculated as WC (cm) divided by the square root of weight (kg) as defined by Park et al [20]. Participants’ WC and body weight information was obtained from body measurements collected at the Mobile Examination Center (MEC).

### 2.4. Assessment of asthma

Based on the methodology of previously published studies, we assessed participants’ asthma status by the Medical Conditions Questionnaire (MCQ) in NHANES [28]. The MCQ is a validated instrument administered by trained interviewers during in-home personal interviews to systematically collect self-reported medical history. For asthma diagnosis, the questionnaire employs a two-step approach:

(1) Participants were first asked: ‘Has a doctor or other health professional ever told you that you have/s/he/Survey Participant (SP) has asthma?’
(2) Those responding affirmatively were then asked: ‘Do you/Does SP still have asthma?’

Current asthma was defined as affirmative responses to both questions. This standardized case definition aligns with CDC surveillance criteria and has been widely adopted in population-based asthma studies [28].

### 2.5. Covariates

By referring to previous relevant literature and clinical experience [29], we included a range of covariates grouped as follows:

Demographic Factors: age, sex, race/ethnicity (non-Hispanic White, non-Hispanic Black, Mexican American, other Hispanic, or other race).
Socioeconomic Status: income-poverty ratio (PIR).
Health and Lifestyle Factors: family history of asthma (assessed via self-report: “Close relative had asthma?”), household smokers (assessed by the question: “Does anyone smoke inside home?”).
Dietary Intake: dietary energy intake (kcal/d), dietary protein, dietary total fat, dietary fiber, dietary vitamin D, dietary calcium, dietary iron, dietary zinc.
Medication Use: anti-asthma medication use (self-reported use of inhaled corticosteroids, leukotriene modifiers, mast-cell stabilizers, and methylxanthines).

### 2.6. Mediating variables

Based on previous literature [13], w we also included several mediating variables that may mediate the association between WWI and asthma prevalence:

Blood Parameters: white blood cell count (WBC), blood eosinophil percentage (EOS%).
Metabolic Markers: gamma-glutamyl transferase (GGT), triglycerides (TG), total cholesterol (TC), high-density lipoprotein cholesterol (HDL-C), and fasting blood glucose (FBG).

The WBC count and EOS% were obtained by complete blood count parameters determined by Beckman Coulter DxH 800. Serum GGT, albumin, TG, TC, HDL-C, and FBG were obtained from the laboratory data.

### 2.7. Statistical analysis

In accordance with NHANES analytic guidelines, we applied appropriate survey weights and accounted for the complex survey design to ensure nationally representative estimates. Participants were analyzed at baseline based on their WWI quartiles and current asthma status. Continuous variables were expressed as mean ± standard error and analyzed using one-way analysis of variance (ANOVA) or t-tests, depending on whether the data were grouped by asthma status or WWI quartiles. Categorical variables were reported as numbers (weighted percentages) and compared using chi-square tests..

Multivariable logistic regression models were performed to investigate independent associations between WWI and asthma prevalence among adolescents, calculating odds ratios (ORs) and 95% confidence intervals (CIs). The crude model did not adjust for any covariates. Model 1 was partially adjusted for age, sex, race, and PIR. Model 2 was fully adjusted and included additional covariates such as family history of asthma, dietary energy intake, dietary protein, total fat, fiber, calcium, iron, zinc, household smoking status, and anti-asthma medication use.

Fully adjusted restricted cubic spline (RCS) models were used to analyze linear or nonlinear dose-response relationships and select appropriate number of knots for smooth curve fitting. In fully adjusted mediation analyses, we explored whether WWI indirectly influenced asthma prevalence among adolescents through mediating variables. The total effect of WWI on the prevalence of asthma consisted of a direct effect of WWI and a proportion mediated through mediating variables [30]. We calculated the mediating proportion of each individual mediating variable in the total effect.

To ensure the robustness of our findings, stratified analyses were conducted based on selected covariates, identifying significant interaction effects. In addition, we performed sensitivity analyses adjusting for BMI (calculated as measured weight in kilograms divided by measured height in meters squared) to determine whether the association between WWI and asthma in adolescents was independent of BMI. We also conducted stratified analyses according to age and sex-specific BMI-defined weight status, categorizing participants as normal (BMI < 85th percentile), overweight (BMI in the 85th-95th percentile), or obese (BMI ≥ 95th percentile). All data processing and statistical analyses were performed using R software (version 4.2.3; R Foundation for Statistical Computing). Statistical significance was defined as a two-tailed p-value < 0.05.

## 3. Results

### 3.1. Baseline characteristics

**Fig 1:**
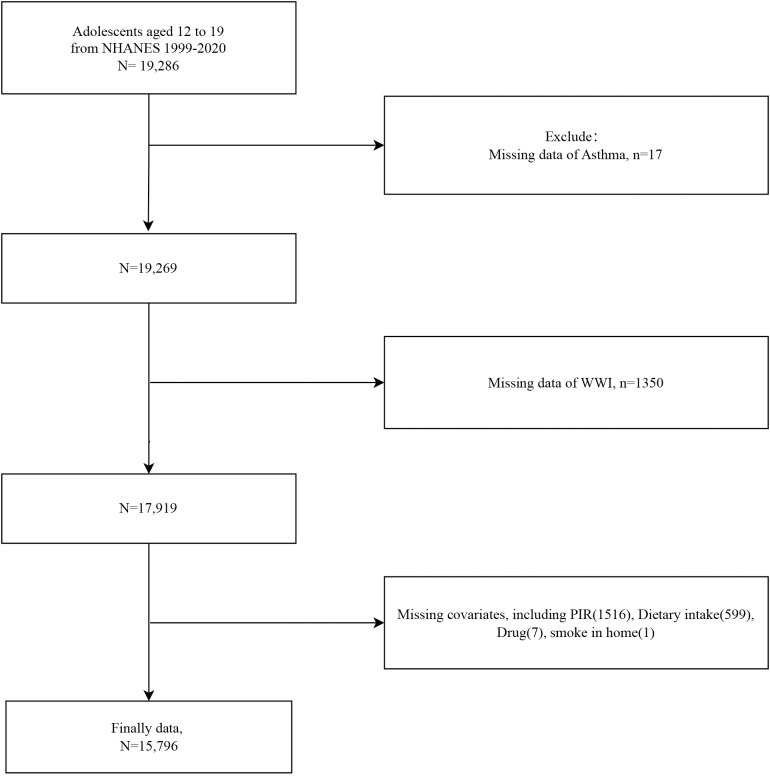
Flowchart of study population selection from NHANES 1999–2020.

We included 19,286 adolescent participants and a flowchart of the participant selection process is provided in Fig 1. Participants were excluded from the study due to missing data, specifically, 17 individuals lacked asthma information, 1,350 were missing WWI diagnostic data, and 2,123 did not have complete covariate data. A total of 15,796 adolescents were finally included, with a mean age of 15.381 years. Baseline analyses based on WWI quartiles (Q1: < 9.574; Q2: 9.574–10.078; Q3: 10.078–10.651; Q4: ≥ 10.651) indicated that participants with higher WWI were younger, had lower PIR, and lower dietary energy, protein, fiber, total fat, calcium, iron, and zinc intake. Additionally, they were more likely to be female, Mexican American, have household smokers, and report current asthma symptoms (Table 1). Participants with current asthma had significantly higher WWI and were more likely to be non-Hispanic Black, use anti-asthma medications, and report a family history of asthma (Table 2).

**Table 1:** Baseline characteristics of adolescent participants according to WWI quartiles.

Variables	Total (n = 15796)	Q1 (n = 3949)	Q2 (n = 3949)	Q3 (n = 3950)	Q4 (n = 3948)	P-value
**Age**	15.381 ± 0.028	15.925 ± 0.045	15.428 ± 0.050	15.187 ± 0.050	14.977 ± 0.056	<0.0001
**PIR**	2.561 ± 0.036	2.814 ± 0.051	2.713 ± 0.052	2.556 ± 0.051	2.124 ± 0.039	< 0.0001
**Dietary energy intake**	2205.403 ± 11.304	2537.738 ± 28.208	2262.783 ± 23.065	2044.569 ± 20.886	1972.688 ± 20.787	<0.0001
**Dietary protein intake**	78.626 ± 0.545	91.559 ± 1.305	82.010 ± 1.063	71.254 ± 0.880	69.531 ± 0.840	<0.0001
**Dietary fiber intake**	14.245 ± 0.118	15.932 ± 0.226	14.502 ± 0.248	13.555 ± 0.170	12.958 ± 0.195	<0.0001
**Dietary fat intake**	83.529 ± 0.539	96.026 ± 1.194	85.644 ± 1.065	78.011 ± 1.006	74.223 ± 0.912	<0.0001
**Dietary calcium intake**	1020.746 ± 9.395	1181.185 ± 17.305	1048.645 ± 18.244	944.344 ± 12.886	906.754 ± 15.000	<0.0001
**Dietary iron intake**	15.385 ± 0.118	17.895 ± 0.279	15.961 ± 0.232	14.085 ± 0.190	13.564 ± 0.185	<0.0001
**Dietary zinc intake**	11.465 ± 0.090	13.508 ± 0.232	11.924 ± 0.177	10.309 ± 0.148	10.104 ± 0.164	<0.0001
**Sex**						<0.0001
male	8033(51.348)	2943(75.315)	2059(52.441)	1532(38.313)	1499(39.674)	
female	7763(48.652)	1006(24.685)	1890(47.559)	2418(61.687)	2449(60.326)	
**Race**						<0.0001
Mexican American	4301(12.725)	433(5.641)	950(10.449)	1303(14.778)	1615(20.456)	
Non-Hispanic Black	4438(13.848)	1955(24.801)	1052(12.472)	744(8.869)	687(9.528)	
Non-Hispanic White	4504(58.879)	1014(57.104)	1246(62.488)	1192(60.732)	1052(54.527)	
Other Hispanic	1097(6.553)	218(5.455)	261(5.900)	331(7.298)	287(7.606)	
Other Race	1456(7.996)	329(6.999)	440(8.690)	380(8.324)	307(7.883)	
**Anti-asthma medication**						0.787
No	15561(98.762)	3899(98.846)	3884(98.873)	3894(98.641)	3884(98.684)	
Yes	235(1.238)	50(1.154)	65(1.127)	56(1.359)	64(1.316)	
**Household smokers**						< 0.0001
No	12218(77.392)	3039(79.840)	3106(79.989)	3109(77.543)	2964(71.677)	
Yes	3578(22.608)	910(20.160)	843(20.011)	841(22.457)	984(28.323)	
**Family history of asthma**						0.284
No	10502(67.072)	2618(67.170)	2659(68.583)	2633(66.426)	2592(65.985)	
Yes	5294(32.928)	1331(32.830)	1290(31.417)	1317(33.574)	1356(34.015)	
**Asthma**						0.015
No	14077(88.832)	3543(90.052)	3542(89.471)	3544(89.047)	3448(86.571)	
Yes	1719(11.168)	406(9.948)	407(10.529)	406(10.953)	500(13.429)	

Continuous variables were expressed as mean ± standard error and examined by one-way analysis of variance (ANOVA); categorical variables were reported as number (weighted percentages) and compared using chi-square tests.

**Table 2:** Baseline characteristics of adolescent participants with and without current asthma.

Variables	Total (n = 15796)	No asthma (n = 14077)	Asthma (n = 1719)	P-value
**WWI**	10.144 ± 0.012	10.133 ± 0.012	10.236 ± 0.031	<0.001
**Age**	15.381 ± 0.028	15.396 ± 0.030	15.264 ± 0.085	0.14
**PIR**	2.561 ± 0.036	2.575 ± 0.037	2.443 ± 0.072	0.063
**Dietary energy intake**	2205.403 ± 11.304	2206.535 ± 11.653	2196.404 ± 35.105	0.78
**Dietary protein intake**	78.626 ± 0.545	78.558 ± 0.536	79.168 ± 1.870	0.746
**Dietary fiber intake**	14.245 ± 0.118	14.263 ± 0.128	14.097 ± 0.273	0.578
**Dietary fat intake**	83.529 ± 0.539	83.591 ± 0.566	83.036 ± 1.654	0.75
**Dietary calcium intake**	1020.746 ± 9.395	1020.680 ± 9.887	1021.274 ± 23.407	0.981
**Dietary iron intake**	15.385 ± 0.118	15.347 ± 0.123	15.694 ± 0.327	0.306
**Dietary zinc intake**	11.465 ± 0.090	11.465 ± 0.093	11.462 ± 0.294	0.991
**BMI**	23.724 ± 0.047	23.555 ± 0.052	24.419 ± 0.112	<0.0001
**WBC**	7.040 ± 0.017	7.019 ± 0.019	7.126 ± 0.042	0.0154
**Eos**	2.997 ± 0.022	2.812 ± 0.023	3.766 ± 0.063	<0.0001
**FBG**	5.253 ± 0.014	5.254 ± 0.016	5.247 ± 0.022	0.818
**GGT**	14.896 ± 0.080	14.734 ± 0.087	15.562 ± 0.197	<0.0001
**TG**	1.077 ± 0.007	1.076 ± 0.007	1.082 ± 0.015	0.719
**TC**	4.129 ± 0.007	4.136 ± 0.008	4.098 ± 0.016	0.0265
**HDL**	1.320 ± 0.003	1.326 ± 0.003	1.295 ± 0.006	<0.0001
**Sex**				0.133
male	8033(51.348)	7152(51.645)	881(48.991)	
female	7763(48.652)	6925(48.355)	838(51.009)	
**Race**				<0.0001
Mexican American	4301(12.725)	3982(13.232)	319(8.692)	
Non-Hispanic Black	4438(13.848)	3810(13.270)	628(18.439)	
Non-Hispanic White	4504(58.879)	3985(58.738)	519(59.997)	
Other Hispanic	1097(6.553)	975(6.646)	122(5.816)	
Other Race	1456(7.996)	1325(8.114)	131(7.056)	
**Anti-asthma medication**				< 0.0001
No	15561(98.762)	13994(99.427)	1567(93.467)	
Yes	235(1.238)	83(0.573)	152(6.533)	
**Household smokers**				0.505
No	12218(77.392)	10934(77.494)	1284(76.581)	
Yes	3578(22.608)	3143(22.506)	435(23.419)	
**Family history of asthma**				<0.0001
No	10502(67.072)	9652(69.463)	850(48.050)	
Yes	5294(32.928)	4425(30.537)	869(51.950)	

Continuous variables were expressed as mean ± standard error and examined by t test; categorical variables were reported as number (weighted percentages) and compared using chi-square tests.

### 3.2. Association of WWI with prevalence of current asthma in adolescents

**Fig 2:**
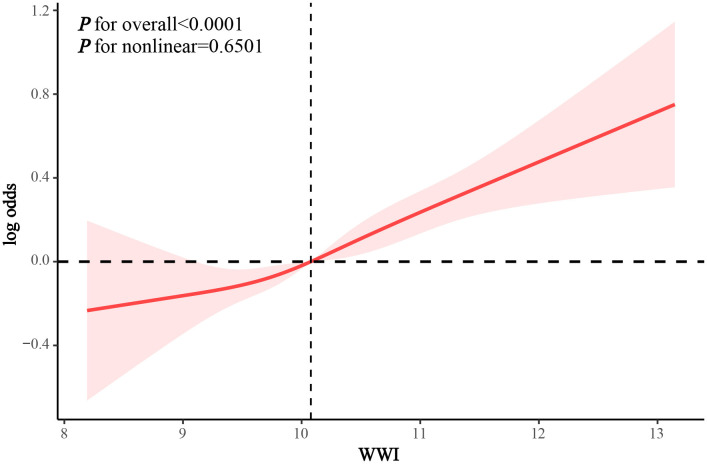
RCS analysis of the association between WWI and odds of asthma in adolescents.

In the crude model and Model 1, we found that WWI was positively associated with the prevalence of asthma in adolescents (OR 1.194 and 1.238, respectively). After adjusting for all confounders, WWI remained positively associated with the prevalence of current asthma among adolescents (OR 1.252, 95% CI 1.125–1.392, p = 0.0001). Compared to Q1, participants with WWI at Q4 had a 54.5% increase in asthma prevalence (95% CI 1.200–1.988; p for trend = 0.0007) (Table 3). RCS analysis demonstrated that WWI exhibited a linear dose-response association with the prevalence of asthma among adolescents (p for nonlinearity = 0.6501) (Fig 2).

**Table 3:** Association between WWI and prevalence of current asthma in adolescents.

	Crude Model OR (95%CI) P-value	Model 1 OR (95%CI) P-value	Model 2 OR (95%CI) P-value
**WWI**	1.194 (1.080, 1.320) 0.0007	1.238 (1.115, 1.374) 0.0001	1.252 (1.125, 1.392) 0.0001
**WWI quartile**			
**Q1**	Ref.	Ref.	Ref.
**Q2**	1.065 (0.833, 1.363) 0.6155	1.118 (0.862, 1.450) 0.4013	1.128 (0.864, 1.473) 0.3785
**Q3**	1.114 (0.882, 1.408) 0.3655	1.194 (0.929, 1.535) 0.1685	1.209 (0.934, 1.564) 0.1517
**Q4**	1.403 (1.116, 1.763) 0.0042	1.506 (1.173, 1.934) 0.0016	1.545 (1.200, 1.988) 0.0009
**P for trend**	0.0045	0.0013	0.0007

The crude model did not adjust for any covariates. Model 1 was partially adjusted for age, sex, race, and PIR. Model 2 was a fully adjusted model that additionally adjusted for family history of asthma, dietary energy intake, dietary protein, dietary total fat, dietary fiber, dietary calcium, dietary iron, dietary zinc, household smokers, and anti-asthma medications from model 1.

### 3.3. Mediation analysis

**Fig 3:**
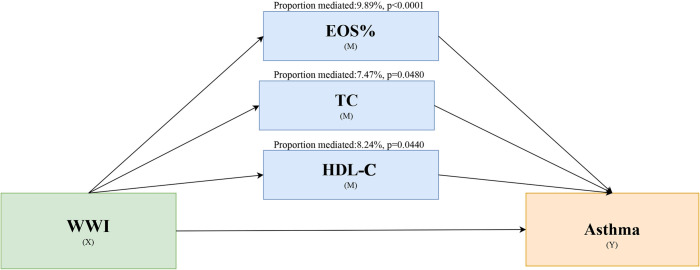
Schematic representation of the effect of WWI on adolescent asthma through mediating variables (EOS%, TC, and HDL-C).

Mediation effect analysis showed significant mediation effects for EOS% (p < 0.0001), TC (p = 0.048), and HDL-C (p = 0.044), whereas there were no significant mediation effects for WBC (p = 0.094), FBG (p = 0.85), GGT (p = 0.352), and TG (p = 0.07) (**Table S1-S7** in S1 File). The proportions mediated by EOS%, TC, and HDL-C in this association were 9.89%, 7.47%, and 8.24%, respectively (Fig 3).

### 3.4. Stratified analysis

**Fig 4:**
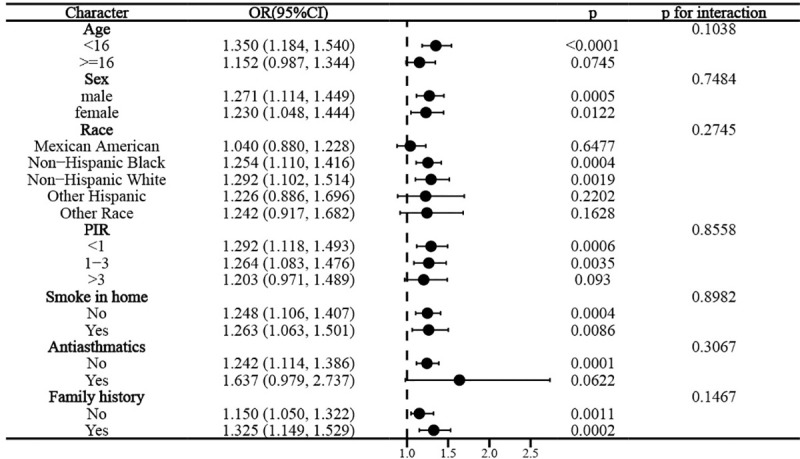
Stratified analysis of the association between WWI and asthma according to selected covariates (Age, sex, race, PIR, family history of asthma, anti-asthma medication use, household smokers).

Interaction tests suggested that this association remained stable across subgroups of age, sex, race, PIR, family history of asthma, household smokers, and anti-asthma medication use (p for interaction all > 0.05), suggesting that the association between WWI and adolescent asthma was not influenced by these factors (Fig 4).

### 3.5. Sensitivity analysis

**Fig 5:**
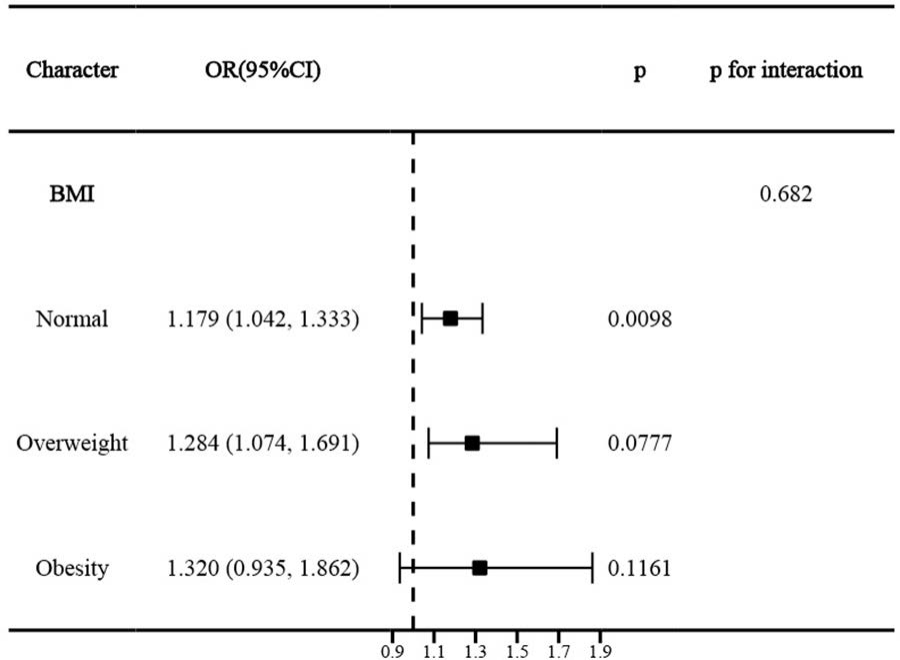
Stratified analysis of the association between WWI and asthma according to BMI in adolescents.

After additional adjustment for BMI based on the fully adjusted model, WWI remained significantly and positively associated with asthma prevalence in adolescents (OR 1.127, 95% CI 1.002–1.268, p = 0.0485). Compared to Q1, participants in the highest quartile had a significant 24% increase in asthma prevalence (p for trend = 0.0003) (Table S8 in S1 File). Stratified analyses based on BMI showed that BMI did not significantly alter the association between WWI and asthma in adolescents (p for interaction = 0.682) (Fig 5).

## 4. Discussion

In a large cross-sectional analysis based on NHANES 1999–2020, WWI was significantly and positively associated with the prevalence of current asthma in U.S. adolescents in a linear dose-response manner. This relationship was notably independent of BMI and remained robust across various confounders. Furthermore, mediation analyses suggest that EOS%, TC, and HDL-C partially explain this association, linking WWI to asthma through inflammatory and metabolic pathways.

To our knowledge, this is the first population-based study to examine the association between WWI and asthma prevalence specifically among adolescents. WWI is recognized as an indicator of central obesity that accurately reflects adult body composition [22–24], with emerging research also exploring its relevance in adolescent populations [31]. Previous studies in adults have already associated higher WWI with increased asthma prevalence, later asthma onset, and even mortality in individuals with asthma [25,26]. Moreover, WWI has demonstrated distinct correlational patterns with asthma duration in adults compared to BMI [27]. Our findings in U.S. adolescents align with this broader literature, demonstrating a consistent positive association between WWI and asthma prevalence. The independence of this association from BMI is crucial, suggesting WWI provides information on asthma risk that is not fully captured by BMI alone. While BMI adjustment slightly attenuated the effect size, WWI remained a significant predictor, underscoring its potential utility in combination with BMI for a more nuanced assessment of asthma risk in adolescents.

Several observational studies have explored the association between BMI and the prevalence of asthma in children and/or adolescents, yet with some ongoing debate. Numerous studies and meta-analyses suggest BMI-defined obesity increases asthma risk [15,29,32,33]. But others report inconsistent associations with asthma outcomes or control, questioning BMI’s utility as a standalone independent risk factor [14,16,34,35]. These inconsistencies, coupled with BMI’s inherent limitation in differentiating fat distribution, have fueled interest in measures of central obesity, like WWI. A meta-analysis that included 13 observational studies showed a significant association between central obesity and increased odds of asthma [45]. A recent study using the NHANES database and Mendelian randomization demonstrated that visceral adipose tissue was an independent risk factor for asthma with a significant causal association [49]. Such indices are hypothesized to better reflect visceral adiposity and its associated pro-inflammatory state, immune dysfunction, and metabolic dysregulation—all pertinent to asthma pathogenesis [13,19,36–41]. Indeed, visceral adipose tissue itself has been identified as an independent, potentially causal risk factor for asthma [42].

Our mediation analysis, identifying EOS%, TC, and HDL-C as partial mediators, supporting the idea that WWI influences asthma risk in adolescents through several interconnected pathways. Specifically, the role of EOS% indicates that central obesity, as measured by WWI, may lead to systemic low-grade inflammation. Visceral fat produces pro-inflammatory cytokines that can exacerbate airway inflammation and hyperresponsiveness, which are critical aspects of asthma [43,44]. Similarly, the mediating roles of TC and HDL-C highlight the contribution of dyslipidemia in the WWI-asthma association [45]. Central obesity often coexists with insulin resistance and an atherogenic lipid profile, characterized by elevated TC and reduced HDL-C. Such lipid alterations may not only impair airway function through increased oxidative stress but could also directly affect airway cell function or alter the production of inflammatory mediators, thus promoting asthma development [46]. Beyond these identified mediators, WWI, as a proxy for central obesity, likely encompasses other pathophysiological processes relevant to asthma. such as mechanical constraints on lung function from excess abdominal fat and the overall influences of metabolic syndrome. These factors highlight WWI’s potential as a valuable indicator for assessing asthma risk in adolescents, complementing traditional measures like BMI. Identifying adolescents with elevated WWI could lead to targeted interventions aimed at reducing asthma risk through lifestyle changes that address central obesity and related metabolic issues.

Our study’s strengths include its large, nationally representative sample, multiethnic population, and comprehensive adjustment for potential confounders, enhancing the reliability of our findings. However, several limitations warrant consideration. First, the cross-sectional design precludes causal inference and leaves potential for residual confounding. Second, current asthma status was based on self-report, introducing potential recall bias. While NHANES protocols instruct interviewers to emphasize that asthma must be doctor-diagnosed, thereby aiming to improve diagnostic accuracy, this remains an inherent limitation of survey-based data [47]. Third, while WWI shows promise, its predictive value for adolescent asthma specifically requires further prospective validation in diverse clinical settings. Finally, the findings are specific to U.S. adolescents, and generalizability to other populations requires further investigation.

## 5. Conclusions

In a large population-based cross-sectional analysis, WWI was positively associated with the prevalence of asthma in US adolescents in a linear dose-response fashion. EOS%, TC, and HDL-C partially mediated this association. This association was independent of and unaffected by BMI and other important confounders. These findings suggest that WWI may serve as an independent predictor of asthma in adolescents and underscore the importance of exploring the real-world predictive value of WWI further in clinical practice.

## Supporting information

S1 File Supplementary materials archive. ZIP file containing: (1) Supplementary Tables S1-S8 (PDF), (2) Asthma study dataset (Excel: asthma_dataset.xlsx), (3) Data analysis code (R script: analysis_code.R).
(ZIP)
