# Supplementary material for: Independent association of weight-adjusted waist index with asthma in U.S. adolescents: Mediating roles of eosinophil percentage, total cholesterol, and HDL cholesterol
Source: PLoS One. 2025 Jul 31;20(7):e0328796. doi: 10.1371/journal.pone.0328796 (PMC12312917; doi:10.1371/journal.pone.0328796)
Supplement: S1 File — ZIP file containing: (1) Supplementary Tables S1-S8 (PDF), (2) Asthma study dataset (Excel: asthma_dataset.xlsx), (3) Data analysis code (R script: analysis_code.R). (ZIP) [file pone.0328796.s001.zip › (1)S1_Table.pdf]

S1 Table. Mediating effects of WBC in the association between WWI and adolescent asthma.

WBC	Estimate	95% CI lower	95% CI upper	*P*-value
Total effect	0.023333	0.015748	0.030452	<0.0001
Mediation effect	0.001751	-0.000261	0.003695	0.0940
Direct effect	0.021583	0.013772	0.029240	<0.0001
Proportion mediated	0.075032	-0.013013	0.173075	0.0940
