# Supplementary material for: Independent association of weight-adjusted waist index with asthma in U.S. adolescents: Mediating roles of eosinophil percentage, total cholesterol, and HDL cholesterol
Source: PLoS One. 2025 Jul 31;20(7):e0328796. doi: 10.1371/journal.pone.0328796 (PMC12312917; doi:10.1371/journal.pone.0328796)
Supplement: S1 File — ZIP file containing: (1) Supplementary Tables S1-S8 (PDF), (2) Asthma study dataset (Excel: asthma_dataset.xlsx), (3) Data analysis code (R script: analysis_code.R). (ZIP) [file pone.0328796.s001.zip › (2)S2_Table.pdf]

S2 Table. Mediating effects of EOS% in the association between WWI and adolescent asthma.

EOS%	Estimate	95% CI lower	95% CI upper	*P*-value
Total effect	0.024183	0.015519	0.031755	<0.0001
Mediation effect	-0.016670	-0.026042	-0.007691	<0.0001
Direct effect	0.025850	0.017300	0.033392	<0.0001
Proportion mediated	0.098925	0.058301	0.136187	<0.0001
