# Supplementary material for: Independent association of weight-adjusted waist index with asthma in U.S. adolescents: Mediating roles of eosinophil percentage, total cholesterol, and HDL cholesterol
Source: PLoS One. 2025 Jul 31;20(7):e0328796. doi: 10.1371/journal.pone.0328796 (PMC12312917; doi:10.1371/journal.pone.0328796)
Supplement: S1 File — ZIP file containing: (1) Supplementary Tables S1-S8 (PDF), (2) Asthma study dataset (Excel: asthma_dataset.xlsx), (3) Data analysis code (R script: analysis_code.R). (ZIP) [file pone.0328796.s001.zip › (4)S4_Table.pdf]

S4 Table. Mediating effects of GGT in the association between WWI and adolescent asthma.

GGT	Estimate	95% CI lower	95% CI upper	*P*-value
Total effect	0.023365	0.015218	0.030554	<0.0001
Mediation effect	0.000843	-0.001046	0.002471	0.3520
Direct effect	0.022522	0.014594	0.030353	<0.0001
Proportion mediated	0.036092	-0.047747	0.121723	0.3520
