# Supplementary material for: Independent association of weight-adjusted waist index with asthma in U.S. adolescents: Mediating roles of eosinophil percentage, total cholesterol, and HDL cholesterol
Source: PLoS One. 2025 Jul 31;20(7):e0328796. doi: 10.1371/journal.pone.0328796 (PMC12312917; doi:10.1371/journal.pone.0328796)
Supplement: S1 File — ZIP file containing: (1) Supplementary Tables S1-S8 (PDF), (2) Asthma study dataset (Excel: asthma_dataset.xlsx), (3) Data analysis code (R script: analysis_code.R). (ZIP) [file pone.0328796.s001.zip › (7)S7_Table.pdf]

S7 Table. Mediating effects of HDL in the association between WWI and adolescent asthma.

HDL	Estimate	95% CI lower	95% CI upper	*P*-value
Total effect	0.023463	0.015586	0.031021	<0.0001
Mediation effect	0.001933	0.000057	0.003925	0.0440
Direct effect	0.021530	0.013212	0.029232	<0.0001
Proportion mediated	0.082389	0.002135	0.190935	0.0440
