# Supplementary material for: Independent association of weight-adjusted waist index with asthma in U.S. adolescents: Mediating roles of eosinophil percentage, total cholesterol, and HDL cholesterol
Source: PLoS One. 2025 Jul 31;20(7):e0328796. doi: 10.1371/journal.pone.0328796 (PMC12312917; doi:10.1371/journal.pone.0328796)
Supplement: S1 File — ZIP file containing: (1) Supplementary Tables S1-S8 (PDF), (2) Asthma study dataset (Excel: asthma_dataset.xlsx), (3) Data analysis code (R script: analysis_code.R). (ZIP) [file pone.0328796.s001.zip › (8)S8_Table.pdf]

S8 Table. Association of WWI with asthma among adolescents after additional adjustment for BMI in the fully adjusted model.

	**OR (95%CI) *P*-value**
WWI	1.127 (1.002, 1.268) 0.0485
WWI quartile	
Q1	Ref.
Q2	1.080 (0.824, 1.414) 0.5786
Q3	1.099 (0.847, 1.425) 0.4790
Q4	1.240 (1.042, 1.632) 0.0069
P for trend	0.0003
